# Non-Canonical Amino Acid-Based Engineering of (*R*)-Amine Transaminase

**DOI:** 10.3389/fchem.2022.839636

**Published:** 2022-02-28

**Authors:** Amol D. Pagar, Hyunwoo Jeon, Taresh P. Khobragade, Sharad Sarak, Pritam Giri, Seonga Lim, Tae Hyeon Yoo, Byoung Joon Ko, Hyungdon Yun

**Affiliations:** ^1^ Department of Systems Biotechnology, Konkuk University, Seoul, South Korea; ^2^ Department of Molecular Science and Technology, Ajou University, Suwon, South Korea; ^3^ School of Biopharmaceutical and Medical Sciences, Sungshin Women’s University, Seoul, South Korea

**Keywords:** (*R*)-amine transaminase, genetic code expansion, enzyme engineering, non-canonical amino acid, *p*-benzoyl-l-phenylalanine

## Abstract

Non-canonical amino acids (ncAAs) have been utilized as an invaluable tool for modulating the active site of the enzymes, probing the complex enzyme mechanisms, improving catalytic activity, and designing new to nature enzymes. Here, we report site-specific incorporation of *p*-benzoyl phenylalanine (*p*BpA) to engineer (*R*)-amine transaminase previously created from d-amino acid aminotransferase scaffold. Replacement of the single Phe88 residue at the active site with *p*BpA exhibits a significant 15-fold and 8-fold enhancement in activity for 1-phenylpropan-1-amine and benzaldehyde, respectively. Reshaping of the enzyme’s active site afforded an another variant F86A/F88*p*BpA, with 30% higher thermostability at 55°C without affecting parent enzyme activity. Moreover, various racemic amines were successfully resolved by transaminase variants into (*S*)-amines with excellent conversions (∼50%) and enantiomeric excess (>99%) using pyruvate as an amino acceptor. Additionally, kinetic resolution of the 1-phenylpropan-1-amine was performed using benzaldehyde as an amino acceptor, which is cheaper than pyruvate. Our results highlight the utility of ncAAs for designing enzymes with enhanced functionality beyond the limit of 20 canonical amino acids.

## Introduction

Chiral amines are valuable and versatile building blocks for the pharmaceutical, agricultural, and fine chemical industries (Mathew and Yun, 2012; Kelly et al., 2018; Patil et al., 2018). Due to the increasing environmental awareness, the development of new biocatalysts to produce optically pure amines is of primary interest (Fuchs et al., 2015; Slabu et al., 2017). Transaminases (TAs), a class of pyridoxal 5′-phosphate (PLP)-dependent enzymes represent an important biocatalysts that provides a green and cost-effective alternative to transition metal catalysts (Guo and Berglund, 2017). Transaminases (TAs) can be classified into two groups: α-transaminases (α-TAs) and ω-transaminases (ω-TAs) depending on the type of substrate that is converted (Steffen-Munsberg et al., 2015). α-TAs requires the presence of a carboxyl group at the α-position with respect to the carbonyl functionality (Rocha et al., 2019). On the other hand, ω-TAs can accept aliphatic ketones and amines as substrates (that is, not only α-keto acids and amino acids). ω-TAs can be further divided into two subgroups, β-TAs, and amine transaminases (ATAs), the latter being commonly used as a synonym for all ω-TAs (Rocha et al., 2019). High enantiospecificity, broad substrate scope, and no need for external cofactor are the beneficial properties of TAs in an industrial context (Steffen-Munsberg et al., 2015). Being PLP-dependent enzymes, TAs can also be classified as (*S*)-selective ATAs belonging to fold-type I (aspartate transaminase superfamily) and (*R*)-selective ATAs belonging to fold-type IV (d-amino acid aminotransferase superfamily) (Slabu et al., 2017). The (*S*)-selective ATAs have been known and widely investigated for industrial use due to their abundance in nature (Łyskowski et al., 2014). In contrast, (*R*)-ATAs have recently been discovered and are less studied than their (*S*)-selective counterparts (Telzerow et al., 2019). The past decade has seen a surge in the interest in (*R*)-ATAs after successfully utilizing engineered (*R*)-ATA-117-Rd11 from *Arthrobacter sp*. KNK168 for the asymmetric synthesis of the antidiabetic drug (*R*)-sitagliptin (Iwasaki et al., 2006; Savile et al., 2010). (*R*)-ATAs catalyze the transfer of an amino group from (*R*)-aromatic or (*R*)-primary aliphatic amines to pyruvate producing ketones or aldehydes and d-Ala (Schätzle et al., 2011; Iwasaki et al., 2012; Sayer et al., 2014; Iglesias et al., 2017; Lakó et al., 2020). Thermodynamically, deamination of amines is preferable; nonetheless, amination of ketones is possible if a sufficient strategy to shift the equilibrium to amine synthesis is utilized (Schätzle et al., 2011; Iglesias et al., 2017; Telzerow et al., 2019, 2021). Several research groups contributed to this field by identification and characterization of new members and employing protein engineering tools to expand the substrate scope and enhance the thermostability (Iwasaki et al., 2006, 2012; Łyskowski et al., 2014; Guan et al., 2015; Hou et al., 2016; Pavkov-Keller et al., 2016; Bezsudnova et al., 2019; Zeifman et al., 2019; Cheng et al., 2020a; Telzerow et al., 2021). For instance, (*R*)-ATA from *Exophiala xenobiotica* has been identified to synthesize biaryl amines, which are considered privileged scaffolds for pharmaceuticals (Telzerow et al., 2019). In parallel, Bornscheuer and colleagues showed the one-pot synthesis of biaryl amine by combining enzymatic and chemical reaction utilizing engineered (*R*)-ATA from *Aspergillus fumigatus* (Dawood et al., 2018)*.* Moreover, by employing an evolutionary approach, substrate scope and thermostability of (*R*)-ATAs from *Mycobacterium vanbaalenii* and *Aspergillus terreus* were enhanced, respectively (Cheng et al., 2020b; Liu et al., 2021). Also, a rationally designed variant V37A of newly characterized (*R*)-ATA from *Luminiphilus syltensis* showed broader substrate scope towards bulkier substrates (Konia et al., 2021). Therefore, it is of interest to identify, characterize and engineer more (*R*)-ATAs to provide broad diversity of applications in the chiral amine synthesis.

(*R*)-ATAs are not the only fold type IV PLP dependent enzymes. (Telzerow et al., 2021). d-amino acid aminotransferases (DATAs), l-branched chain aminotransferases (BCATs), and 4-Amino-4-deoxychorismate lyases (ADCLs) hold an overall structural similarity, therefore, belong to the same family (Telzerow et al., 2021). Still, their amino acid sequence, activity, stereo preference, and substate scope considerably differ (Bezsudnova et al., 2020). ADCLs catalyze the production of *p*-aminobenzoate from 4-amino-4-deoxychorismate as part of folate biosynthesis (Dai et al., 2013). On the other hand, the specificity of DATAs, BCATs is concerned mainly about α-amino and α-keto acids while (*R*)-ATAs concerns (*R*)-primary amines (Bezsudnova et al., 2020). Unlike (*S*)-ATAs, class IV TAs are inert against polyamines and β-, γ-, and ω-amino acids (Bezsudnova et al., 2020). Besides their function, these different subfamilies can also be distinguished by the sequence-based motifs assigned by Bornscheuer and co- workers (Höhne et al., 2010). These motifs have advanced the identification and characterization of several new class IV TAs, thereby providing valuable insights about this fold type (Kelly et al., 2020). Among them (*R*)-ATAs are of particular interest due to their ability to synthesize enantiomerically pure (*R*)-amines from respective ketones (Ferrandi and Monti, 2018). Thus, it is essential to extend the knowledge of this important enzyme class by identifying and characterizing new members and structure-guided rational design approaches, thereby supporting protein engineering efforts to expand the toolbox of these industrially valuable enzymes (Bezsudnova et al., 2020)., To this end, Voss et al. demonstrated an evolutionary approach for the (*R*)-ATA from DATA using bioinformatic analysis combined with a computational redesign. A sextuple variant (Y31F/H86F/Y88F/H100L/S180A/T242I) was obtained with a specific activity of 326 mU mg^−1^ in the conversion of (*R*)-phenylethylamine (**1a**) (Voss et al., 2020). This study paves an alternate route to expand the toolbox and deepens our understanding of the class IV transaminases.

Although, protein engineering techniques such as rational design and directed evolution have found widespread applications in improving or altering the intrinsic activities of numerous enzymes (Arnold, 2018; Chen and Arnold, 2020). These strategies are primarily based on nature’s alphabet of twenty canonical amino acids (cAAs) (Qu et al., 2020; Wang et al., 2021). However, less than half of cAAs have side chains with functional groups involved in enzymes' catalytic mechanisms (Green et al., 2016; Yu et al., 2021). Because of the limited chemical and physical repertoire of cAAs, not surprisingly, enzymes recruit reactive cofactors and post-transnationally modify existing amino acids in the active site (Yu et al., 2018). The advancements in the genetic code expansion (GCE) allow enhanced protein properties by introducing unique functional groups beyond nature’s limited building blocks (Wang and Schultz, 2004; Young and Schultz, 2018). To this end, a series of orthogonal amino-acyl transfer RNA (tRNA) synthetase (aaRS)/tRNA pairs have been developed to encode distinct non-canonical amino acids (ncAAs) *in vivo* (Chin, 2014, 2017; Young and Schultz, 2018; Tseng et al., 2020; Koch et al., 2021; Lee et al., 2021). Over the last 2 decades, more than 200 ncAAs have been genetically encoded in prokaryotes and eukaryotes (Pagar et al., 2021). Apart from peptide modification, antibody development for pharmaceutical use, ncAAs has been widely applied in enzyme engineering research to illustrate the enzyme mechanisms, enhance enzyme activity, and even generate new catalytic mechanisms into protein scaffolds (Agostini et al., 2017; Won et al., 2019b; Drienovská and Roelfes, 2020; Giri et al., 2021; Pagar et al., 2021). The examples include ncAAs with metal chelating, photo-crosslinking, extended disulfide forming, orthogonal reactive, and unique pi-pi interaction properties (Pagar et al., 2021). Although progress in this field is fast, only a handful of examples have been reported for remodelling enzyme active sites by introducing single ncAA resulting in improved activity and substrate scope compared to cAAs (Pagar et al., 2021). The major bottlenecks for the practical utilization of ncAAs in enzyme engineering are lower expression yields, high cost or unavailability of the ncAAs, background incorporation of cAAs, and limited structural diversity of ncAAs utilizing only orthogonal tyrosyl/pyrrolysyl-tRNA synthetases (Agostini et al., 2017; Drienovská and Roelfes, 2020). Nonetheless, continued efforts to overcome the above limitations open new doors in enzyme engineering and serve as a toolkit for evolution and designing enzymes with desired functionality (Pagar et al., 2021). In this work, we report ncAA-based engineering of (*R*)-ATA previously created from DATA scaffold. The rational incorporation of ncAA has significantly improved enzyme functionality. These results uncover the great potential of engineering enzymes with ncAAs for the efficient synthesis of chiral amines.

## Materials and Methods

### Materials

The plasmids pDule-tfmF A65V S158A (ID: 85484) (Miyake-Stoner et al., 2010) and PylRS-AS (ID:137908) (Lee et al., 2016) were purchased from Addgene (Watertown, MA, United States). The gene for (*R*)-ATA and primers were synthesised and sequenced by BIONICS Co., Ltd. (Seoul, South Korea). The 2,3,4-triflourophenylalanine (F3F) was purchased from ChemImpex Inc (Wood Dale, IL, United States), *p*BpA and *p*-methylphenylalanine (*p*MeF) were purchased from BACHEM (Bubendorf, Switzerland), and *p*-trifluoromethylphenylalanine (*p*tFMF) from Alfa Aesar. Ni–NTA affinity columns were purchased from Qiagen (Valencia, CA, United States). All the other chemicals like pyruvate, acetophenone, pyridoxal 5′-phosphate and amino donors were purchased from Sigma-Aldrich, Korea.

### Site Directed Mutagenesis

The plasmid pET24ma harboring a gene for (*R*)-ATA was utilized as a template for inserting TAG codon as well as Ala mutations. The list of primers used is given in Supplementary Table S1. The thermal cycler was programmed as 1) initial denaturation at 95°C for 2 min, 2) 18 cycles of denaturation at 95°C for 30 s, annealing at 58°C for 30 s (depending on the *T*_m_ of the primers) and extension at 72°C for 5 min; 3) a final extension at 72°C for 5 min (Sarak et al., 2021). The resulting PCR product was digested with DpnI for 2 h at 37°C and transformed into chemically competent *E. coli* DH5-alpha cells. A single colony was picked from LB plates supplemented with Kanamycin and cultured overnight in 5 ml of LB media. The plasmids were isolated, and the gene was sequenced to confirm desired mutation.

### ncAA Incorporation and Purification of the Mutant Enzymes

The plasmid pET24ma harboring the gene encoding for (*R*)-ATA and the TAG codon variants were individually co-transformed with PylRS-AS or pDule-tfmF A65V S158A or pEVOL-*p*BpARS (Park et al., 2018) into chemically competent *E. coli.* (BL-21) cells. A single colony of host cells was incubated overnight into 5 ml LB-media supplemented with respective antibiotics. The selected ncAAs were incorporated into target enzyme by the reported protocols for each ncAAs (Chin et al., 2002; Jackson et al., 2007; Miyake-Stoner et al., 2010; Lee et al., 2016). The expression of *p*BpARS and F_3_FPylRS was induced by 0.2% arabinose and expression of target enzyme was induced by 0.1 mM IPTG. The overexpressed cells were harvested by centrifugation at 5,000 rpm, 4°C for 10 min. The pellet was suspended in an appropriate volume of the lysis buffer (NaH_2_PO_4_ (50 mM), NaCl (300 mM), imidazole (5 mM, pH 8.0)). The cell suspension was then subjected to ultrasonic disruption by a horn-type sonicator (Sonics& Material Inc, United States). During sonication, the sample tube containing the cell mass suspension was held in an ice bath. The sonication was carried for the total duration of 30 min, with a duty cycle of 37.5%. The cell lysate was centrifuged at 17,000 rpm for 30 min and supernatant was loaded on Ni-NTA agarose resin column [BabyBio] with the flow rate of 1 ml/min. The non-target proteins were washed with 100 ml of washing buffer (50 mM NaH_2_PO_4_, 300 mM NaCl and 20 mM imidazole, pH 8.0) with flow rate of 2 ml/min. Next, the desired protein containing C-terminal hexa-His-tag was eluted using elution buffer 1 (50 mM NaH_2_PO_4_, 300 mM NaCl and 250 mM imidazole, pH 8.0). The eluted solution containing the purified protein was dialyzed against 20 mM Tris-HCl buffer (pH 8.0) and concentrated using an Amicon 30K ultrafiltration unit.

### Determination of Enzyme Activity

All enzyme assays were carried out at 37°C and 100 mM Tris-HCl buffer (pH-9.0). Standard substrate conditions for activity assay were 10 mM (*R*)-1a, 10 mM pyruvate and 0.1 mM PLP. The typical reaction volume was 500 μL, and the enzyme reaction was stopped after 30 min by adding 500 μL 10% perchloric acid. Acetophenone produced was analysed by HPLC. Here, different concentrations of enzymes were used to measure the exact specific activity.

### Determination of Substrate Specificity

Amino acceptor specificity was measured by using 10 mM (*R*)-1a as an amino donor and 10 mM amino acceptor at 37°C and 100 mM Tris Hcl buffer (pH-9.0) for 30 min. Acetophenone produced was analysed by HPLC. Amine substrate specificity was measured using 10 mM pyruvate and 10 mM respective amine compound (1a-1e) solubilized using 10% DMSO. Respective ketone product formed in each reaction after 30 min was analyzed by HPLC.

### Analytical Conditions

Acetophenone and other ketone products were analyzed using a C_18_ Symmetry column (Agilent) with an elution mixture of 50% methanol (0.1% TFA) and 50% water (0.1% TFA) at a flow rate of 1.0 ml/min at 244 nm detection wavelength. The quantitative analysis of amines was measured using HPLC with a Crownpak CR (Daicel Co., Japan) column at 210 nm with an elution of pH 1.5 perchloric acid solution (0.6 ml min^−1^).

## Results and DISCUSSION

### Incorporation of ncAAs Into (*R*)-ATA

**FIGURE 1:**
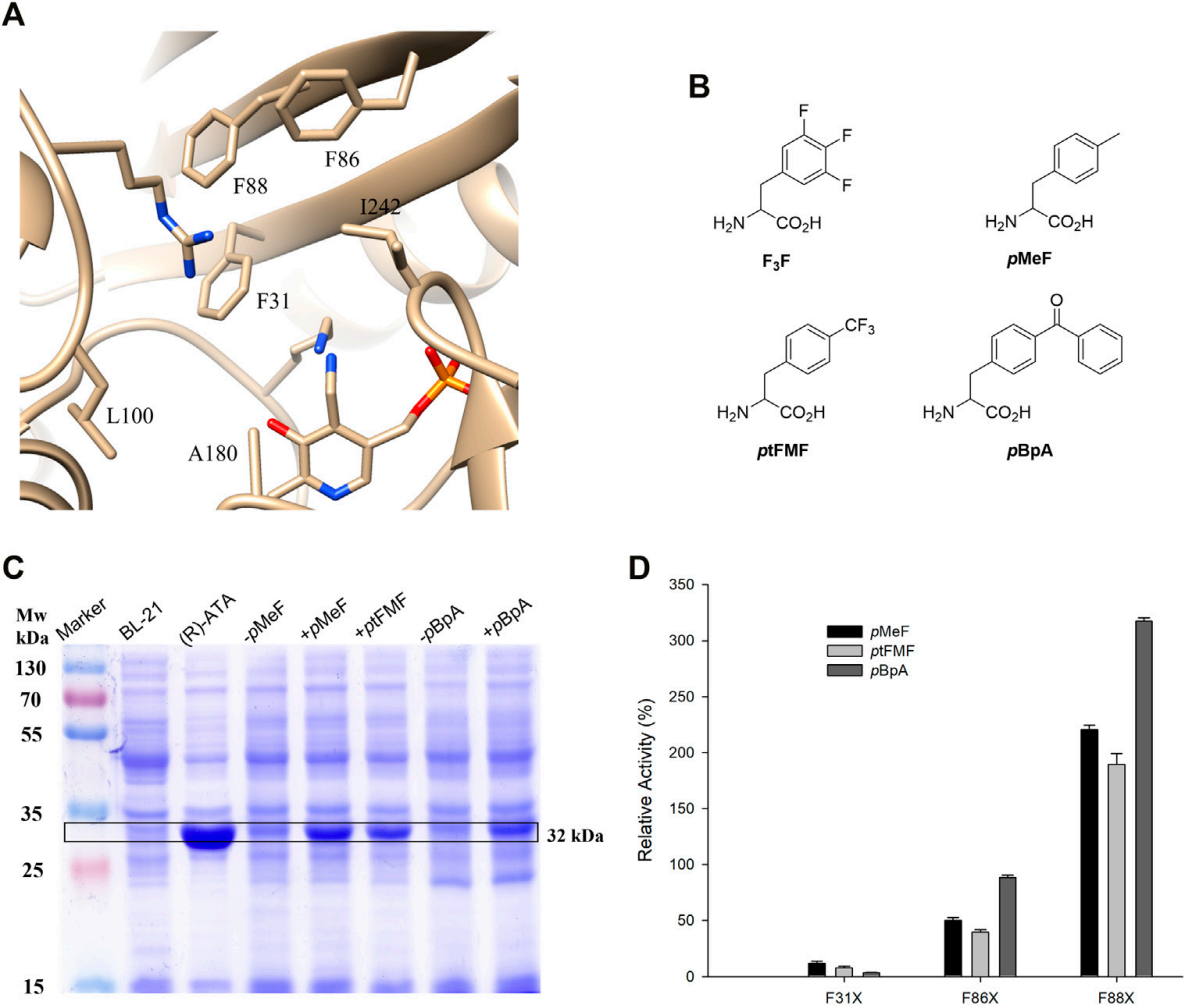
**(A)** Active site residues of rationally designed (*R*)-ATA from DATA scaffold. **(B)** Structures of ncAAs selected for incorporation into active site of (*R*)-ATA **(C)** Coomassie-stained SDS-PAGE showing expression of proteins in presence and absence of ncAAs; **(D)** Relative activities of variants containing three selected ncAAs at 31^st^, 86^th^ and 88^th^ position. The specific activity of parent (*R*)-ATA (324 mU mg^−1^) was taken as 100%.

Class IV TAs are promising catalysts for synthesizing enantiomerically pure amines and d-amino acids (Bezsudnova et al., 2020). The development of (*R*)-ATA for manufacturing sitagliptin demonstrated the practical viability of (*R*)-ATAs for asymmetric synthesis on an industrial scale (Savile et al., 2010). It sparked new research in the field of (*R*)-ATAs in synthetic applications. Yet, narrow ketone substrate range, unfavourable reaction equilibria, and strong ketone inhibition limit the applicability of these enzymes (Steffen-Munsberg et al., 2015; Guo and Berglund, 2017). In conjunction with identifying new TAs, structure-guided rational design approaches could help overcome these bottlenecks (Steffen-Munsberg et al., 2015). To this end, Voss et al. studied two members from fold type IV, BCAT and DATA, and their respective variants to accept the benchmark amine substrate 1a. By employing bioinformatic analysis combined with the computational redesign, a DATA variant (M2-6) harbouring six mutations (Y31F/H86F/Y88F/H100L/S180A/T242I) (Figure 1A), was generated yielding a total activity of 326 mU mg^−1^ toward 1a. A significant increase in active site volume by non-polar side chain mutations contributed to the favourable binding of bulkier and hydrophobic 1a over d-alanine. Despite bearing moderate (*R*)-ATA activity, the rational engineering of DATA deepens our knowledge of how substrate specificity in α-ATA is affected and can be altered toward accepting arylamines. Notably, three among six mutations introduced were Y31F/H86F/Y88F, vastly contributing to the active site’s hydrophobicity. The rational design approach based on cAAs limits to create a more hydrophobic environment into the active site as Phe is highly hydrophobic among all cAAs. The GCE strategies allow the incorporation of more than 200 ncAAs with different functional properties into proteins (Won et al., 2019b; Pagar et al., 2021). Therefore, we speculate that substitution of Phe by a ncAA with a more hydrophobic side chain could enhance the activity of this rationally designed enzyme. Initially, we selected four Phe analogs, namely, F_3_F, *p*tFMF, *p*-methylphenylalanine *p*MeF, and *p*BpA, based on commercial availability and hydrophobicity of the side chains (Figure 1B). An amber stop codon was substituted for three individual Phe residues (F31, F86, and F88). Initially, the incorporation efficiencies of selected ncAAs were determined by incorporating each ncAA at the 31^st^ position using available orthogonal tRNA synthetase/tRNA pairs evolved for each ncAA (Supplementary Table S2). The incorporation efficiency of F_3_F using evolved *Methanosarcina mazei* pyrrolysyl-tRNA synthetase/tRNA pair was comparatively poor (Supplementary Figure S1), and media shift, high concentration of ncAA (5 mM) longer expression time, is quite unpractical for obtaining higher concentrations of the mutant enzyme. The tRNA synthetase/tRNA pair evolved from *Methanococcus jannaschii* tyrosyl tRNA synthetase for *p*MeF, *p*tFMF and *p*BpA incorporation showed better incorporation efficiency and fidelity (Figure 1C). Therefore, total nine mutants containing each of three ncAAs at 31^st^, 86^th^ and 88^th^ position were purified along with their parent (*R*)-ATA and subjected to an activity assay, which was conducted using 10 mM 1a and 10 mM pyruvate in 100 mm Tris/HCl buffer (pH 9.0). One unit was defined as the amount of enzyme that catalysed the formation of 1 µmol of acetophenone per minute. Incorporation of ncAAs at 31^st^ position showed deleterious effects on the TA activity, implying F31 is highly conserved. Remarkably, replacing F88 with all three ncAAs displayed higher activities than the parent enzyme with highest 3-fold activity by F88pBpA (Figure 1C). This enhanced activity was in close agreement with the previous finding where replacement of the Y88 with Phe showed a beneficial effect on (*R*)-PEA acceptance (Voss et al., 2020). On the other hand, replacing F86 with *p*MeF and *p*tFMF showed ∼50 and 61% reduction in activity, respectively, while pBpA hardly affected the enzyme activity.

### Reshaping of the Active Site

**FIGURE 2:**
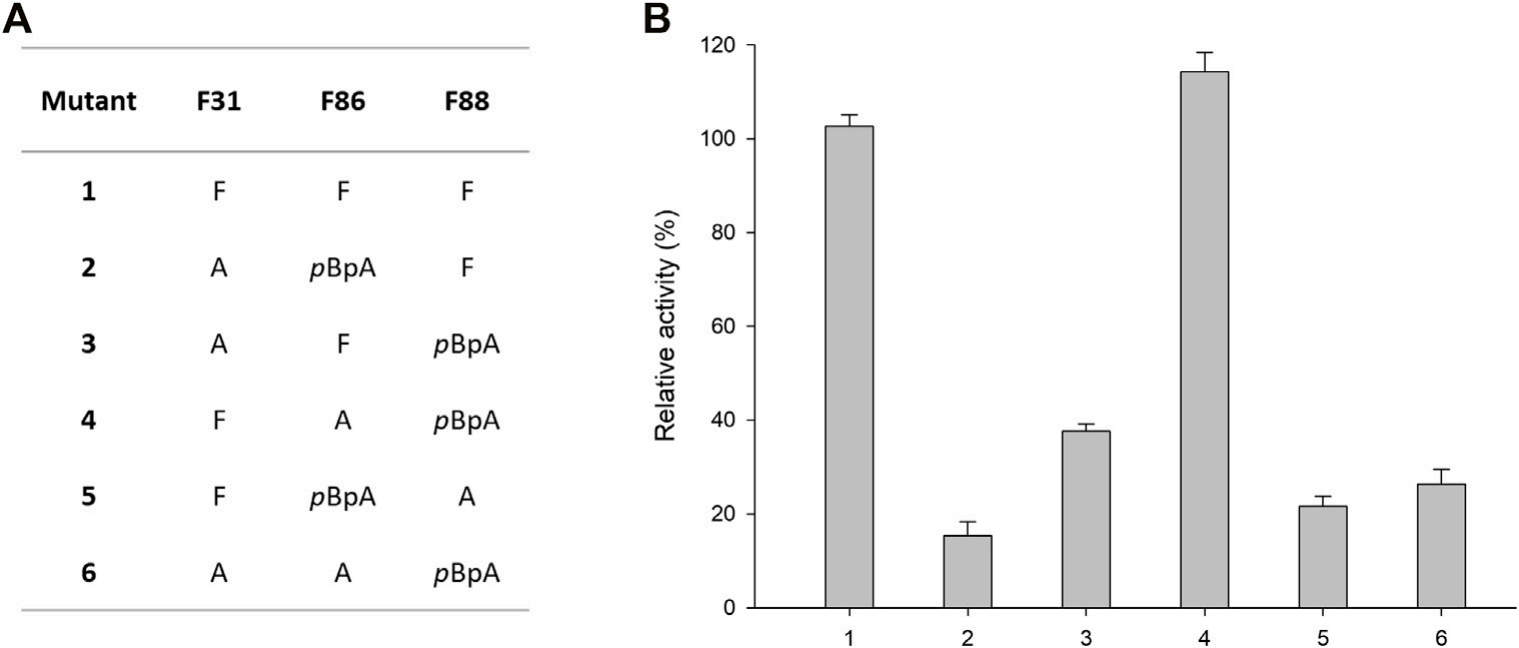
Alanine scanning of the active site of (*R*)-ATA **(A)** Mutations introduced into active site of (*R*)-ATA having one *p*BpA and another Phe mutated to Ala. **(B)** The relative activity of the *p*BpA/Ala mutants towards 1a. The specific activity of the parent (*R*)-ATA (324 mU mg^−1^) was taken as 100%.

Encouraged by the above results, we further aimed to reshape the active site of (*R*)-ATA with ncAA. Three Phe residues provide enough hydrophobicity to the active site but significantly compromise the substrate-binding pocket’s size and thus narrow the substrate scope. Therefore, we envisioned that the hydrophobicity provided by single *p*BpA introduced into the active site might compensate for Phe residue’s replacement. Ala and Phe have hydrophobic side chains, but the earlier is much smaller and more flexible than the latter. Therefore, an Ala scan was performed on three selected Phe residues while retaining one residue as *p*BpA. Mutant combinations with F31*p*BpA were omitted owing to the detrimental effect of *p*BpA substitution at 31st position (Figure 1C). Total five mutants were designed (Figure 2A), and activities of the resulting variants were measured against 1a. Significant reductions in enzyme activity toward 1a were observed, apparently due to the impairment of the hydrophobic environment required to recognize the phenyl group of 1a (Figure 2B). In contrast, variant F86A/F88*p*BpA showed activity comparable to the parent enzyme, suggesting F31 and F88 residues play an essential role in recognizing the phenyl ring of 1a. Additionally, the loss of hydrophobicity by replacing F86 with Ala was compensated by *p*BpA, thereby retaining the overall enzyme activity. The variant F86A/F88*p*BpA was taken for further characterization.

### Substrate Specificity of the Designed Variants

**FIGURE 3:**
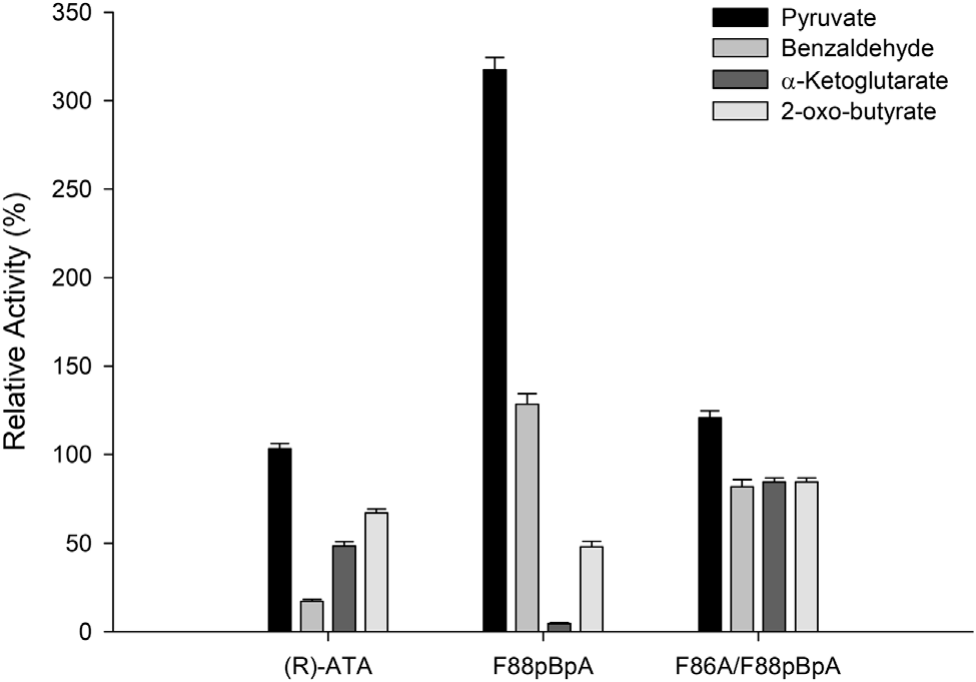
Substrate specificities of the (*R*)-ATA and its variants towards various amino acceptors. (*R*)-ATA activity towards pyruvate (324 mU mg^−1^) was taken as 100%.

Next, the substrate specificities of parent (*R*)-ATA, F88*p*BpA, and F86A/F88*p*BpA were investigated using a series of commonly used amino acceptors like pyruvate, benzaldehyde, α-ketoglutarate, and 2-oxobutyrate, using 1a as an amino donor. Pyruvate served as the best amino acceptor for all the variants, and F88*p*BpA showed a specific activity of 1.03 U mg^−1^. Interestingly, F88*p*BpA and F86A/F88*p*BpA showed ∼8- and 5-fold higher activities respectively, for benzaldehyde than the parent enzyme (Figure 3). This increased activity could be attributed to the enhanced acceptance of aromatic substrates by *p*BpA incorporation. As a cheaper amino acceptor than pyruvate, benzaldehyde can be suitable for TA reactions. In contrast, the activity of F88*p*BpA variant towards α-ketoglutarate has been drastically reduced while F86A/F88*p*BpA retained its activity towards all amino acceptors.

**FIGURE 4:**
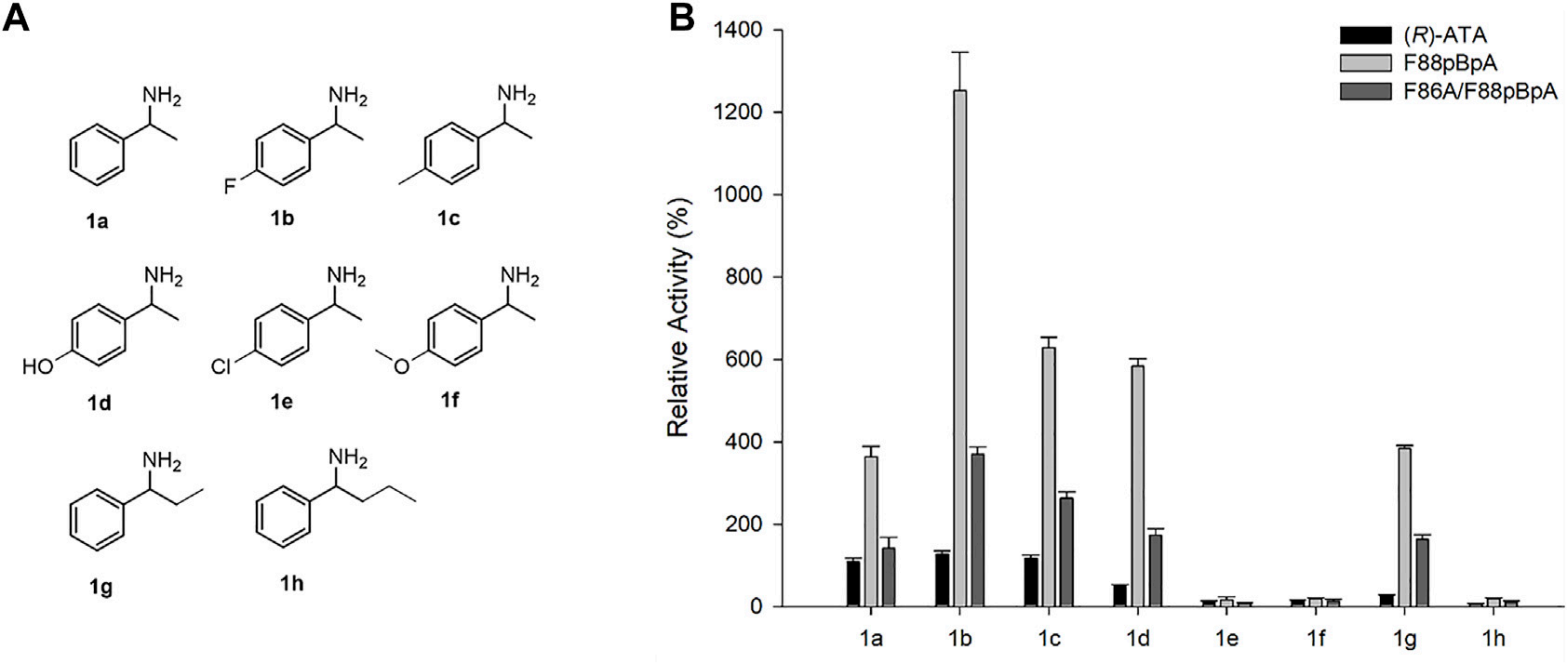
Amino donor specificity of selected (*R*)-ATA mutants **(A)** Substrate structures and **(B)** Relative activity. Different concentrations of the enzyme were used to determine specific activity exactly. Specific activity of (*R*)-ATA for 1a (324 mU mg^−1^) was taken as 100%.

Moreover, the substrate specificities of variants were examined for various substituted arylamines (1a-h) as shown in Figure 4A using pyruvate as an amino acceptor. The respective ketone product formed in 10 mM reaction was quantified by HPLC. While parent enzyme showed mediocre activities towards 1a-d and 1g, F88*p*BpA and F86A/F88*p*BpA showed significantly enhanced activities (Figure 4B). Notably, 1g carrying a 26% parental reactivity relative to 1a showed the highest ∼15-fold enhancement in activity upon F88*p*BpA substitution. Notably, parent (*R*)-ATA and its variants were able to accept ethyl side chain into P-pocket (small-binding pocket) which is uncommon among most of the naturally occurring (*R*)-ATAs (Cheng et al., 2020b; Konia et al., 2021). In addition, ∼12-fold enhanced activity for 1d which is carrying *p*-hydroxy substitution, suggests that even a polar substituted aryl amine’s acceptance has been improved by F88*p*BpA substitution. Unfortunately, none of the mutant showed activity for 1e, 1f and 1h. F86A/F88*p*BpA was expected to have a broader substrate scope due to enlarged active site by F86A mutation. Nevertheless, more active site remodelling efforts are still necessary to improve the substrate scope of this rationally designed enzyme.

### Effect of pBpA Incorporation on Stability

**FIGURE 5:**
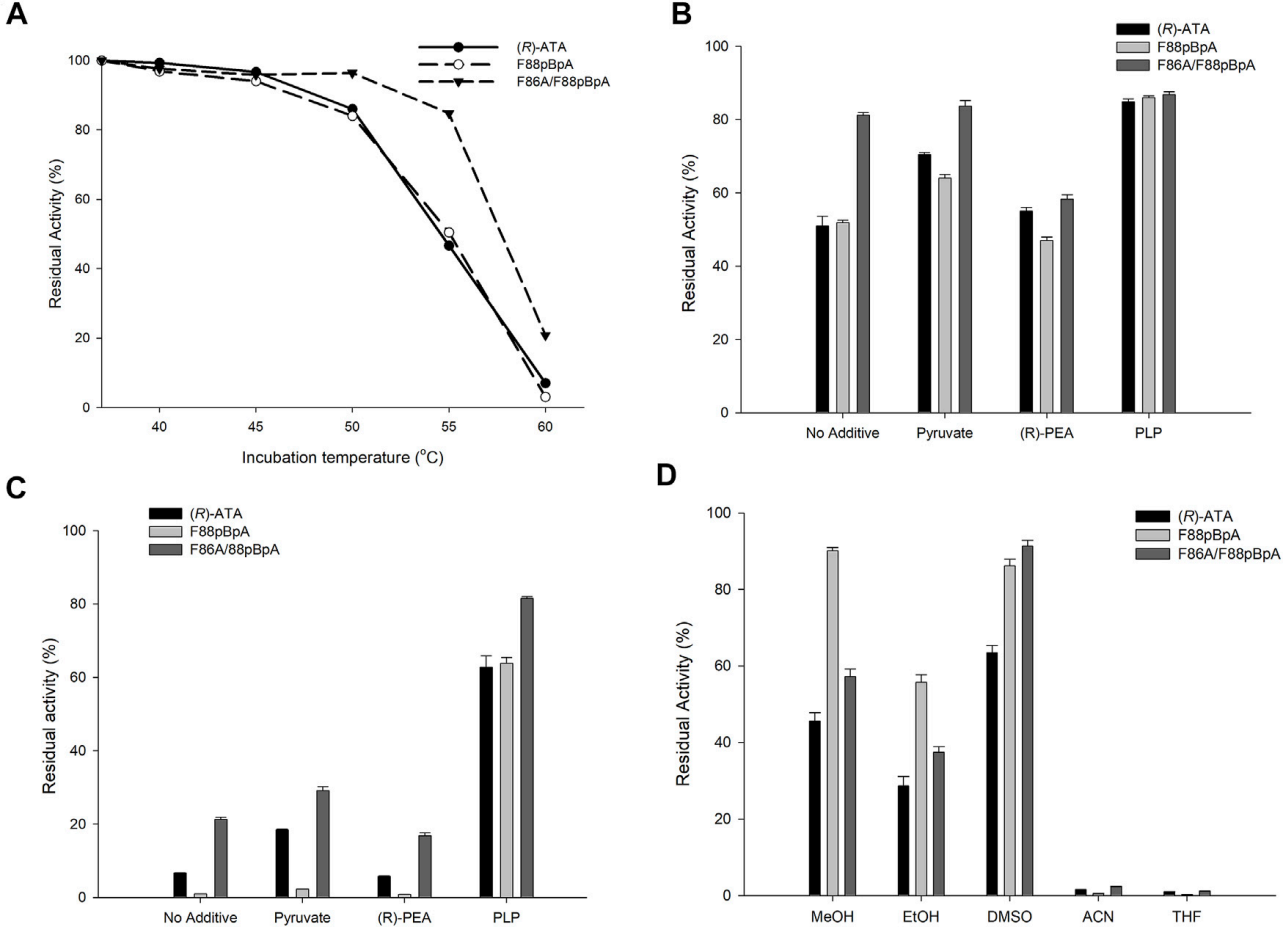
**(A)** The residual activity of (*R*)-ATA and its variants at 37–60°C without any additive. The residual activity of (*R*)-ATA and its variants in the presence of pyruvate, **1a,** and PLP at **(B)** 55°C, and **(C)** 60°C. **(D)** The residual activity of (*R*)-ATA and its variants in the presence of different organic solvents (20% v/v).

It has been observed that improved activity or substrate specificity of enzymes leads to a trade-off between stability in many instances (Siddiqui, 2017; Yu and Dalby, 2018). However, it is worth mentioning that the (*R*)-ATA utilized herein was previously created from the DATA from *Bacillus subtilis* without affecting overall stability (Voss et al., 2020). Incorporation of halogenated ncAAs by selective pressure incorporation or site-specific incorporation in several instances has enhanced the stability of enzymes (Hoesl et al., 2011; Deepankumar et al., 2014; Carlsson et al., 2018; Ohtake et al., 2018; Won et al., 2019a). Nevertheless, incorporation of single *p*BpA has not yet been reported for improved stability. Therefore, we were curious whether incorporating *p*BpA into (*R*)-ATA affected the stability. To this end, we examined the residual activity of the variants by incubating enzyme samples at various temperatures (37–60°C) in the presence of 100 mM Tris HCl (pH-9.0) for 30 min (Figure 5A). Unfortunately, the most active variant F88*p*BpA and the parent enzyme lost almost 45% of their intrinsic activity after incubating at 55°C, while F86A/F88*p*BpA retained 85% of its original activity. These results demonstrate that enhanced activity does not necessarily lead to a trade-off with stability as best variant F88*p*BpA showed a similar stability profile with the parent enzyme. On the other hand, F86A/F88*p*BpA showed greater thermostability than the parent enzyme, indicating that it is indeed possible to tune the activity and stability profiles of the enzymes using ncAAs. Moreover, the thermal stability of enzyme variants was examined under different conditions because 1a diminishes stability due to formation of E-PMP (Deepankumar et al., 2014; Mathew et al., 2016). In contrast, pyruvate and PLP have a beneficial effect on stability. The residual activities of enzymes were further examined after 30 min of incubation in 100 mM Tris/HCl buffer (pH-9.0) at 55 and 60°C and in the presence of 10 Mm 1a, 10 mM pyruvate, or 0.1 mM PLP. Interestingly parent (*R*)-ATA and F88*p*BpA regain their stability at 55°C in presence of PLP displaying 83% overall activity similar to F86A/F88*p*BpA (Figure 5B). Next, the enzyme activities were significantly decreased at 60°C in presence of 1a and without additive, whereas PLP showed stabilizing effect by forming E-PLP complex. Variant F86A/F88*p*BpA retained highest 81% of activity where native enzyme and F88*p*BpA lost ∼35% of their original activity at 60°C (Figure 5C). Owing to the poor solubility of keto or amine substrates in aqueous buffer solutions, organic solvents are generally used as a co-solvent. Therefore, the stability of enzyme variants was also examined in presence of various organic solvents (20% v/v) like methanol, ethanol, DMSO, acetonitrile and tetrahydrofuran. All the variants showed better stability after incubation in presence of DMSO and methanol whereas, complete loss of activity was observed in presence of acetonitrile and tetrahydrofuran (Figure 5D). In agreement with enhanced stability, F86A/F88*p*BpA also showed better organic solvent tolerance in presence of 20% DMSO than other variants.

### Kinetic Resolution of Amines

Incorporation of ncAAs into enzymes enables a dramatic expansion of their catalytic features. However, the biocatalytic use of enzymes containing ncAAs lacks behind the use of ncAAs as tools for therapy and research (Pagar et al., 2021). Low expression levels, high cost or unavailability of ncAAs, unfolded enzymes, incompletely translated enzymes and background incorporation of the cAAs are the major bottlenecks for the GCE method from being a standard method for enzyme engineering (Drienovská et al., 2020). Expression yield of the protein containing ncAA also reflects the degree by which mutation is tolerated (Kolev et al., 2014). For instance, the expression yields of F88*p*BpA and F86A/F88*p*BpA were 32–35 and 68–70 mg/L respectively whereas parent (*R*)-ATA co-expressed with *p*BpARS yielded ∼134–140 mg/L of protein. Also, affinity purification is rather unpractical for screening of large set of mutants or large scale biocatalytic process. Therefore, we performed the kinetic resolution of racemic amines using whole cell despite the fact that expression levels of soluble enzymes harboring *p*BpA will be less than parent (*R*)-ATA (Figure 1C). Since, pyruvate was a good amino acceptor for all variants, the kinetic resolution of 20 mM 1a-1d and 1g was carried out with equimolar concentration of pyruvate (Supplementary Figure S2–6). As shown in Table 1 after 24 h reaction, 50% conversion of 1g with >99% *ee* was achieved by F86A88*p*BpA whereas, F88*p*BpA and parent (*R*)-ATA showed only 45 and 36% conversion, respectively. Mutant F86A/F88*p*BpA performed well than F88*p*BpA for all the substrates perhaps due to its higher stability and expression levels. For the kinetic resolution of amines, it is necessary to add stoichiometric quantities of pyruvate which is used as an amino acceptor. However, pyruvate itself is an expensive compound and thus increases the overall cost of the biocatalytic production of amines (Shin et al., 2015). Interestingly, F88*p*BpA showed considerable reactivity towards benzaldehyde (Figure 3) as 463 mU mg^−1^ suggesting that very cheap benzaldehyde can be used as a good amino acceptor. Therefore, kinetic resolution of 10 mM 1g was performed using 20 mM benzaldehyde. Highly enantiomerically pure (*S*)-1g with 50% conversion and >99% *ee* was obtained by parent (*R*)-ATA and its variants (Supplementary Figure S7). This result clearly demonstrates the applicability of mutant TAs using benzaldehyde as a cheap amino acceptor in a kinetic resolution reaction system for producing optically pure amines.

**TABLE 1:** Kinetic resolution of various amine compounds using parent (*R*)-ATA and its variants.

Substrate	Parent (*R*)-ATA		F88*p*BpA		F86A/F88*p*BpA	
	Conversion (%)	*ee* (%)	Conversion (%)	*ee* (%)	Conversion (%)	*ee* (%)
1a	22	28	17	21	27	38
1b	50	>99	44	78	50	>99
1c	38	60	36	57	43	76
1d	34	52	41	70	40	68
1 g	36	56	45	83	50	>99
1 g*	50	>99	50	>99	50	>99

Reaction conditions: Reaction Vol. 1 ml. 20 mM rac-(1a-1d and 1g), 20 mM pyruvate, 1.98 mg_CDW_/mL E. coli, 0.1 mM PLP, 100 mM Tris-HCl buffer (pH-9.0) at 37°C. *kinetic resolution of 10 mM rac-1g using 20 mM benzaldehyde as an amino acceptor instead of pyruvate.

The kinetic resolution is not an efficient way to utilize (*R*)-ATAs for synthesizing (*S*)-amines. Several (*S*)-selective ATAs can be employed to produce (*S*)-enantiomers with a theoretical yield up to 100% by asymmetric synthesis (Mathew and Yun, 2012; Slabu et al., 2017; Kelly et al., 2018; Patil et al., 2018). Therefore, we tried to perform (*R*)-ATA catalyzed asymmetric synthesis of (*R*)-1g from propiophenone using benzylamine as an amino donor. Though enzymes utilized in this study were reactive for deamination of (*R*)-1g, they could not perform the reverse reaction, i.e., amination of the propiophenone. This indicates that, unlike most of the ω-TAs, enzyme variants utilized herein do not follow the “good donor-acceptor pair relationship” (Han et al., 2017). However, we believe that F88*p*BpA or F86A/F88*p*BpA may serve as a good template for protein engineering to obtain efficient biocatalysts to produce bulky (*R*)-amines.

## Discussion

ATAs are the PLP dependent enzymes which catalyze the transfer of an amino group from an amino donor to a carbonyl moiety and vice versa (Steffen-Munsberg et al., 2015). Transaminase reactions gain popularity for the production of chiral amines because of their outstanding optical purity, high yield, broad substrate specificity, and environmental friendliness (Patil et al., 2018). On account of their importance in asymmetric biocatalysis, the identification and characterization of new members and engineering efforts to improve their functional properties are of utmost importance (Steffen-Munsberg et al., 2015).

Transaminase classification has recently been revised (Gao et al., 2017). To date, only a few (*R*)-ATA are identified, and their crystal structures were made available, allowing researchers to get insight into the enantioselectivity and explore the catalytic mechanism compared to their (*S*)-selective counterparts (Konia et al., 2021). Furthermore, researchers used molecular modeling and the structure-activity relationship to create the enzymes (Voss et al., 2020). However, problems like an unfavorable equilibrium constant and the inability to accept bulky substrates still need to be addressed (Bezsudnova et al., 2020). Although engineering (*R*)-ATA from *Arthrobacter sp*. was a noteworthy, the rational quest for more relevant (*R*)-ATAs is still ongoing because it will lay the groundwork for these enzymes’ future applications (Savile et al., 2010).

The chemical modification and ncAA incorporation methods have emerged as an important alternative to the traditional enzyme engineering approaches like directed evolution and rational design (Giri et al., 2021; Pagar et al., 2021). These methods used independently or together have dramatically expanded the chemical diversity for proteins, which has provided protein engineers with powerful tools for enzymes engineering (Pagar et al., 2021). Over the past few decades, numerous studies have reported increased enzyme activity and stability by incorporating the ncAAs (Agostini et al., 2017; Drienovská and Roelfes, 2020). In a pioneering example, Jackson and others demonstrated that site-specific incorporation of ncAAs can be used to redesign the enzyme’s active site for diverse substrates, resulting in a ∼30-fold increased activity that cannot be reproduced by substituting any other cAA (Jackson et al., 2006).

In this study, we demonstrated the ncAA-based engineering approach to enhance the functionality of rationally designed (*R*)-ATA. Based on the preliminary knowledge that hydrophobicity is a major contributing factor for the creation of (*R*)-ATA activity, we explored it further using ncAA incorporation. The designed variant containing *p*BpA at 88^th^ position has significantly enhanced the activity towards several arylamine substrates. Moreover, another engineered variant F86A/F88*p*BpA showed enhanced thermostability and organic solvent tolerance. These results clearly demonstrate that a wise selection of ncAAs for rational engineering of the enzymes can render beneficial results for functional enhancement. However, further engineering efforts are still needed to improve the substrate scope of this enzyme. To this goal, simultaneous incorporation of ncAAs using mutually orthogonal aminoacyl tRNA synthetases/tRNA pairs for the incorporation of distinct ncAAs into one protein could be beneficial (Neumann et al., 2010; Italia et al., 2019). Moreover, directed evolution with ncAAs can also be utilized to enlarge the chemical and sequence space of proteins and, in turn, increase the probability of evolving the desired mutant (Pagar et al., 2021; Pan et al., 2021).

The main obstacle in the ncAA-based enzyme engineering and directed evolution approach is exogenous supplementation of often expensive ncAA into the growth medium (Pagar et al., 2021). This increases the overall production cost of the ncAA containing enzymes. Autonomous biosynthesis of ncAAs and their concurrent incorporation into enzyme of interest *in vivo* could significantly reduce the production cost and permeability issues (Pagar et al., 2021). Using enzymatic and metabolic pathways, some ncAAs like *p*-amino-phenylalanine, 5-hydroxytryptophan, l-phosphothreonine, l-dihydroxyphenylalanine, fluorotyrosine, and *S*-allylcysteine were biosynthesized in *E. coli* and concurrently incorporated into target proteins (Ma et al., 2014; Exner et al., 2017; Kim et al., 2018; Won et al., 2019a; Nojoumi et al., 2019; Schipp et al., 2020). The production of an increasing number of ncAAs in engineered cells will be aided by advances in biochemistry, molecular biology, and synthetic biology. In addition, advancements in ncAA mutagenesis procedures may drive ncAAs more valuable in protein engineering and enzyme evolution (Pagar et al., 2021).

## Data Availability

The original contributions presented in the study are included in the article/Supplementary Material, further inquiries can be directed to the corresponding author.
