# Supplementary material for: Non-Canonical Amino Acid-Based Engineering of (R)-Amine Transaminase
Source: Front Chem. 2022 Feb 28;10:839636. doi: 10.3389/fchem.2022.839636 (PMC8918476; doi:10.3389/fchem.2022.839636)
Supplement: Supplementary file 1 [file DataSheet1.pdf]

# *Supplementary Material*

## Codon optimized nucleotide sequence of (*R*)-ATA

ATGGGCTATACCCTGTGGAATGATCAGATTGTGAAAGATGAAGAAGTGAAAATTGATAAAGAAGATCGTG
GCTATCAGTTTGGCGATGGCGTGTTTGAAGTGGTGAAAGTGTATAATGGCGAAATGTTTACCGTGAATGAA
CATATTGATCGTCTGTATGCGAGCGCGGAAAAAATTCGTATTACCATTCCGTATACCAAAGATAAATTTCAT
CAGCTGCTGCATGAACTGGTGGAAAAAAATGAACTGAATACCGGCTTTATTTTTTTTCAGGTGACCCGTGG
CACCAGCCCGCGTGCGCTTCAGTTTCCGGAAAATACCGTGAAACCGGTGATTATTGGCTATACCAAAGAAA
ATCCGCGTCCGCTGGAAAATCTGGAAAAAGGCGTGAAAGCGACCTTTGTGGAAGATATTCGTTGGCTGCGT
TGCGATATTAAAAGCCTGAATCTGCTGGGCGCGGTGCTGGCGAAACAGGAAGCGCATGAAAAAGGCTGCT
ATGAAGCGATTCTGCATCGTAATAATACCGTGACCGAAGGCAGCGCCAGCAATGTGTTTGGCATTAAAGAT
GGCATTCTGTATACCCATCCGGCGAATAATATGATTCTGAAAGGCATTACCCGTGATGTGGTGATTGCGTGC
GCGAATGAAATTAATATGCCGGTGAAAGAAATTCCGTTTACCACCCATGAAGCGCTGAAAATGGATGAACT
GTTTGTGACCAGCACCATTAGCGAAATTACCCCGGTGATTGAAATTGATGGCAAACTGATTCGTGATGGCA
AAGTGGGCGAATGGACCCGTAAACTTCAGAAACAGTTTGAAACCAAAATTCCGAAACCGCTGCATATTTCA
GGAGGATCC

**Table S1. Oligonucleotide primers used in this study.**

| Primer | Oligonucleotide sequence |
|---|---|
| Nde I (F) | ATTA CATATG GGCTATACCCTGTGGAATG |
| Xho I (R) | ATTA CTCGAG GGATCCTCCTGAAATATG |
| F31TAG (F) | GATGGCGTGTAGGAAGTGGTG |
| F31TAG (R) | CACCACTTCCTACACGCCATC |
| F86TAG (F) | AATACCGGCTAGATTTTTTTT |
| F86TAG (R) | AAAAAAAATCTAGCCGGTATT |
| F88TAG (F) | CCGGCTTTATTTAGTTTCAGGTGACC |
| F88TAG (R) | GGTCACCTGAAACTAAATAAAGCCGG |
| F31A (F) | GATGGCGTGGCTGAAGTGGTG |
| F31A (R) | CACCACTTCAGCCACGCCATC |
| F86A/F88TAG (F) | CTGAATACCGGCGCTATTTAGTTTCAG |
| F86A/F88TAG (R) | CTGAAACTAAATAGCGCCGGTATTCAG |
| F86TAG/F88A (F) | ACCGGCTAGATTGCTTTTCAGGTGACC |
| F86TAG/F88A (R) | GGTCACCTGAAAAGCAATCTAGCCGGT |

**Table S2. Plasmids used in this study**

| Vector | Function | Reference |
| --- | --- | --- |
| pET24ma-(*R*)-ATA | Expression of target enzyme | This Study |
| pEVOL- PylRS-AS | Incorporation of $F_3F$ | (Lee et al., 2016) |
| pDule-tfmF A65V S158A | Incorporation of *p*MeF and *pt*FMF | (Miyake-Stoner et al., 2010) |
| pEVOL-*p*BpARS | Incorporation of *p*BpA | (Chin et al., 2002; Park et al., 2018) |

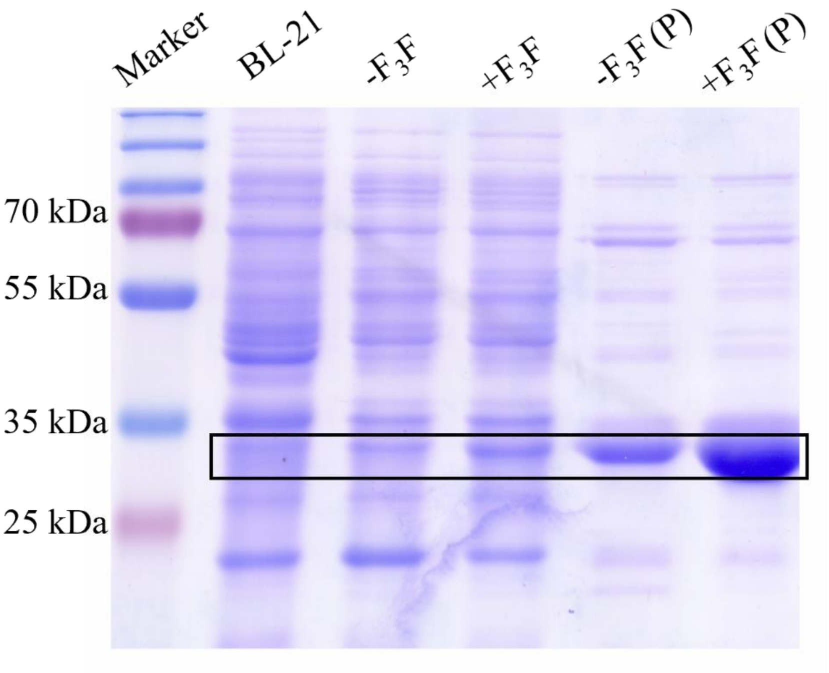


**Figure S1.** Coomassie-stained SDS-PAGE showing expression of (*R*)-ATA in presence and absence of $F_3F$. (P: purified enzyme)

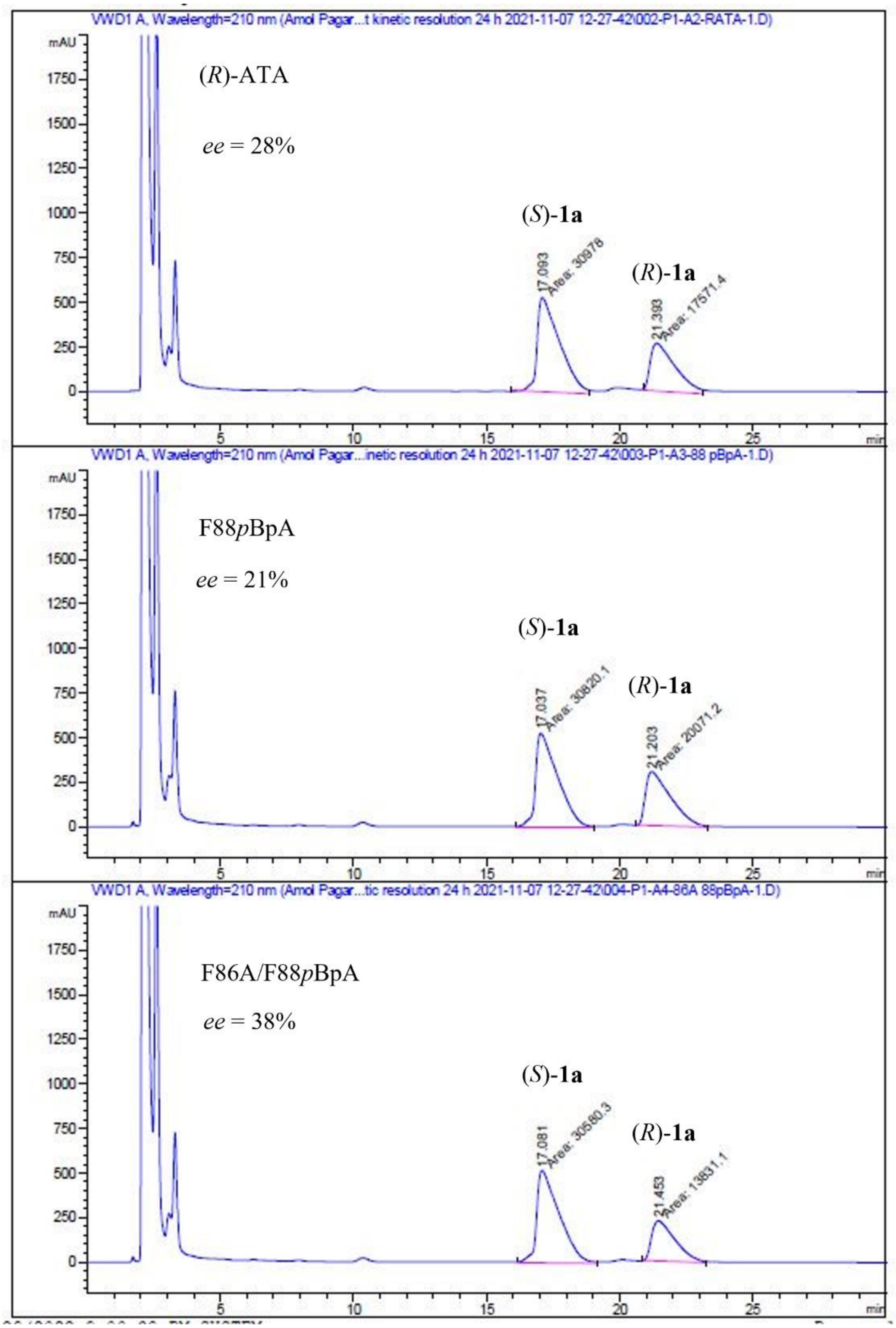


**Figure S2**: HPLC chromatogram of the kinetic resolution of 20 mM *rac*-**1a** using (*R*)-ATA and its variants.

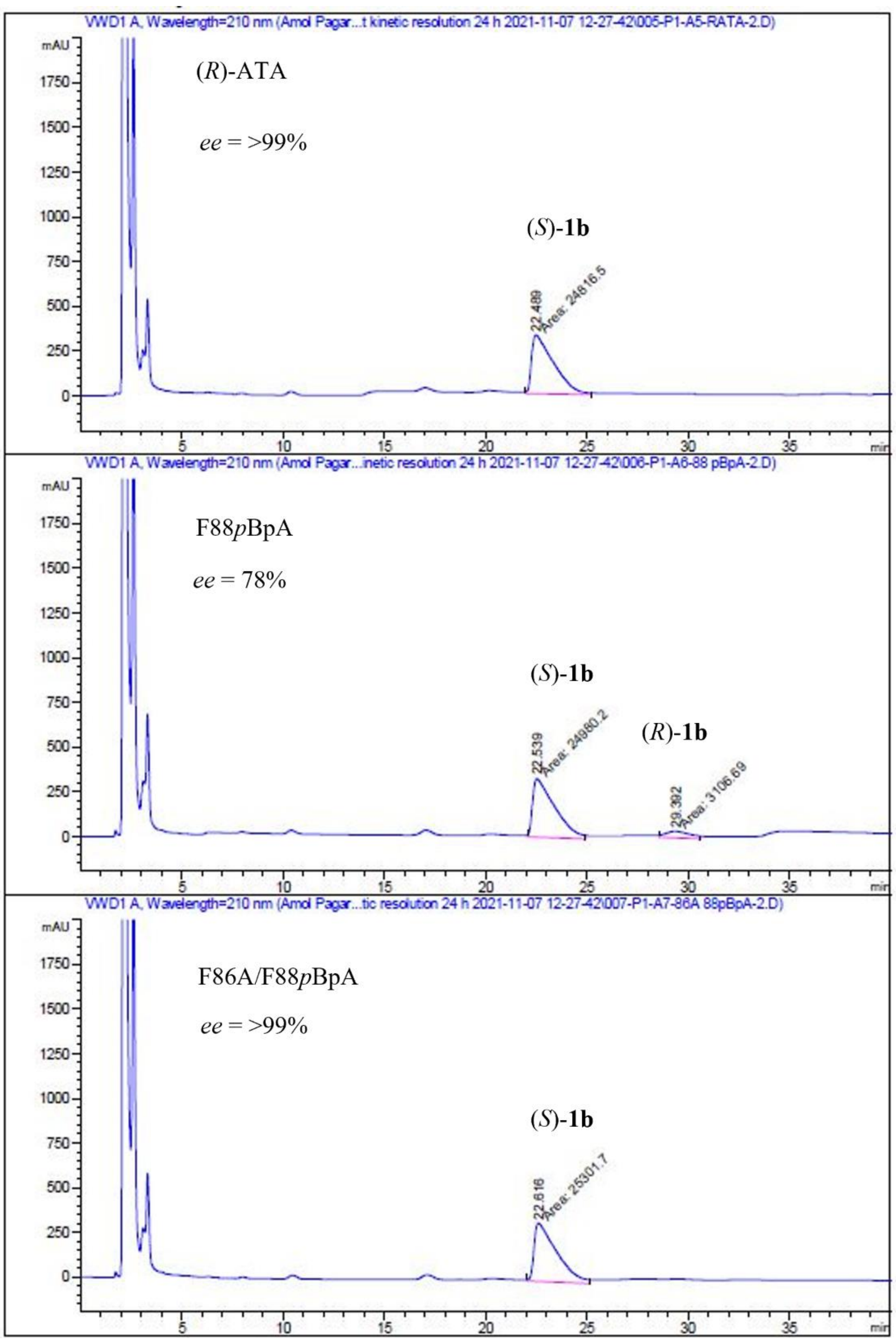


**Figure S3:** HPLC chromatogram of the kinetic resolution of 20 mM *rac*-**1b** using (*R*)-ATA and its variants.

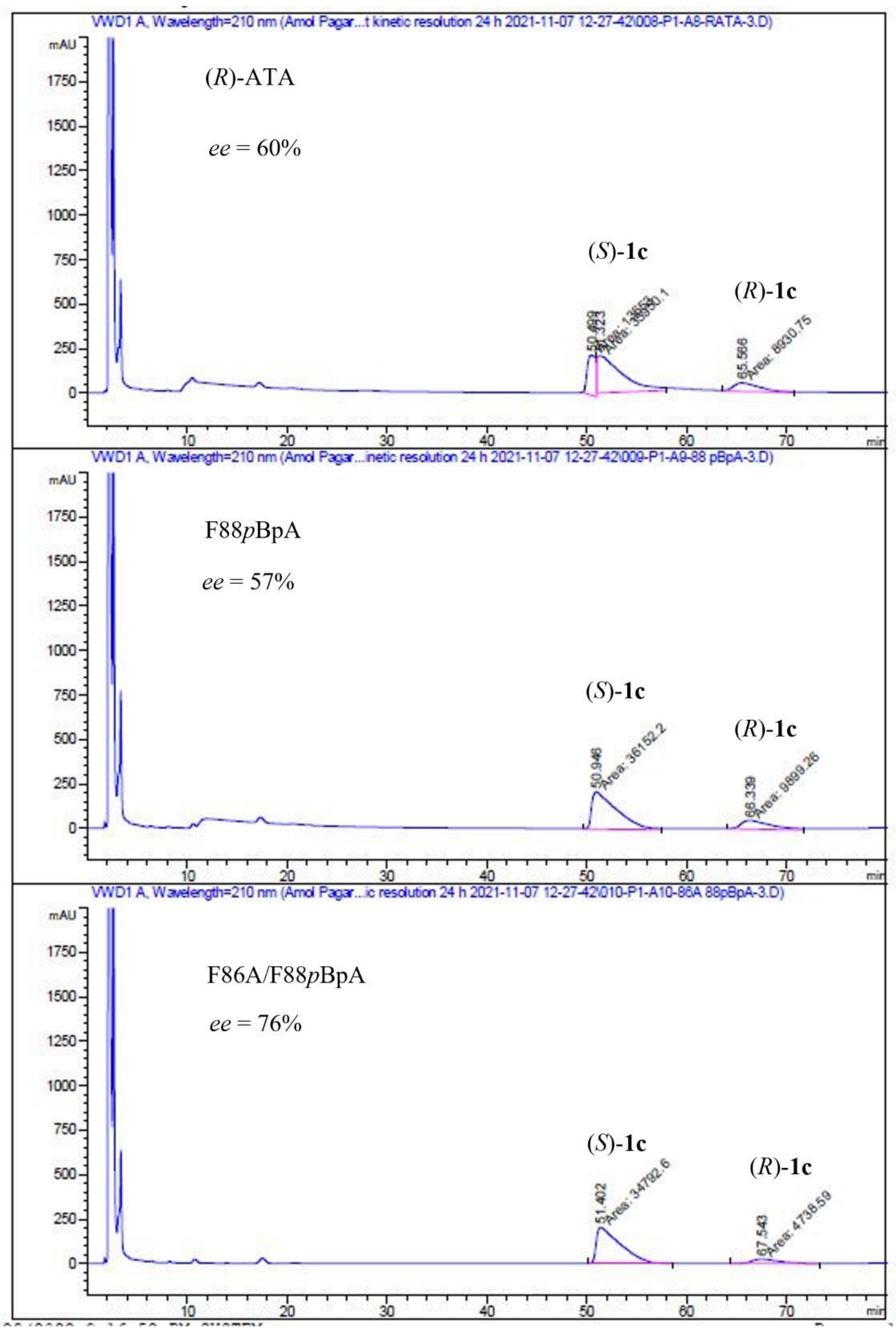


**Figure S4:** HPLC chromatogram of the kinetic resolution of 20 mM *rac*-**1c** using (*R*)-ATA and its variants.

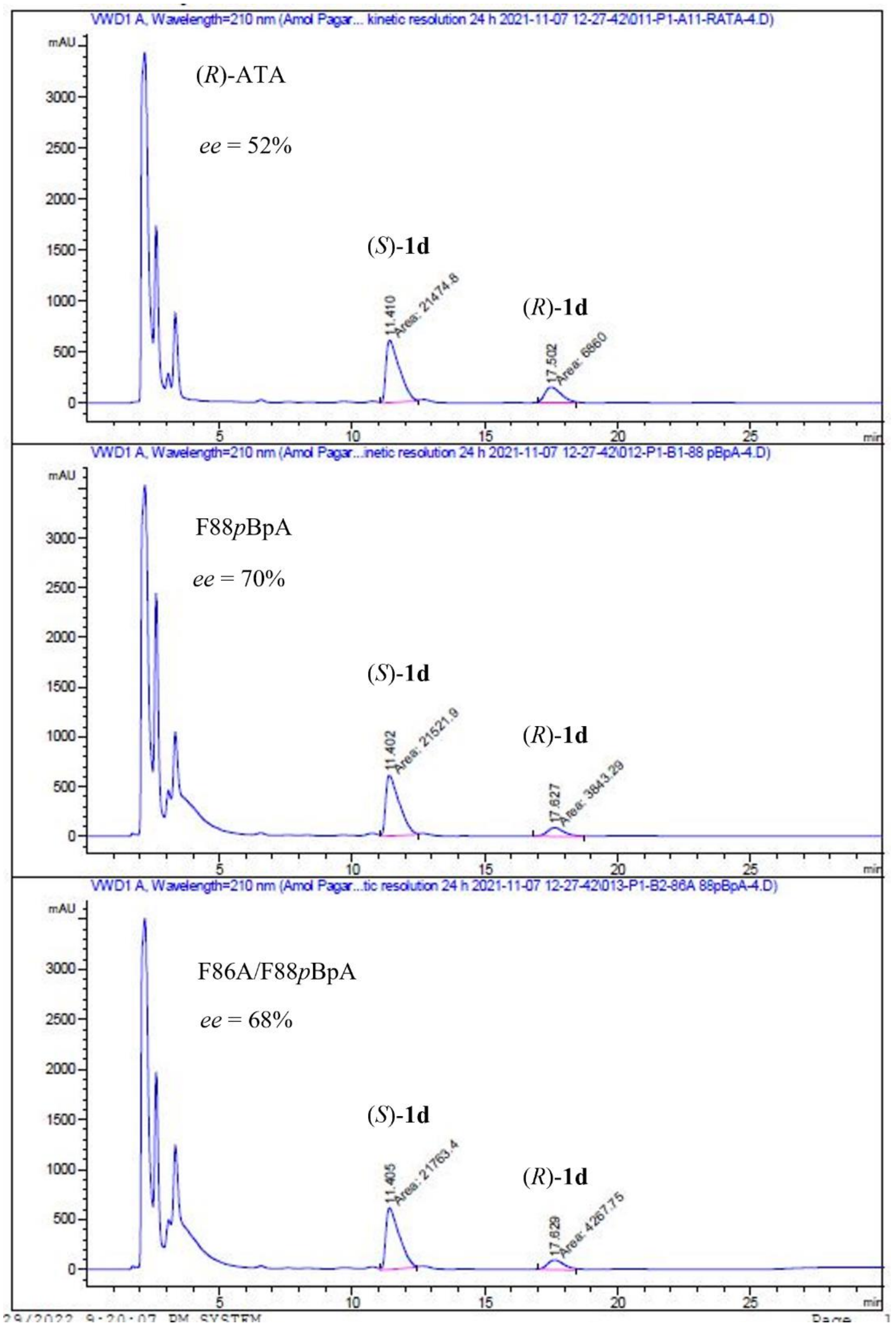


**Figure S5:** HPLC chromatogram of the kinetic resolution of 20 mM *rac*-**1d** using (*R*)-ATA and its variants.

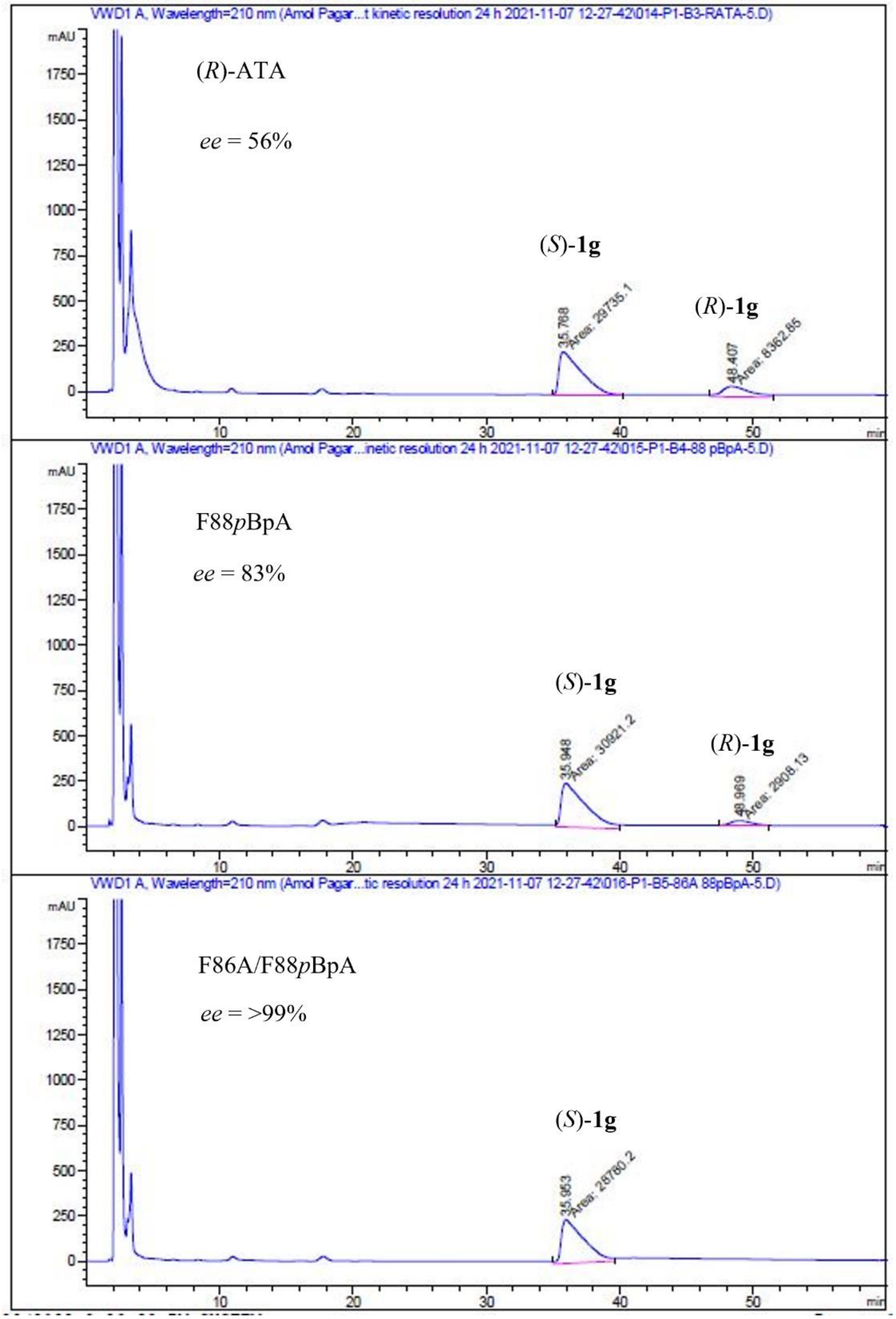


**Figure S6:** HPLC chromatogram of the kinetic resolution of 20 mM *rac*-**1g** using (*R*)-ATA and its variants.

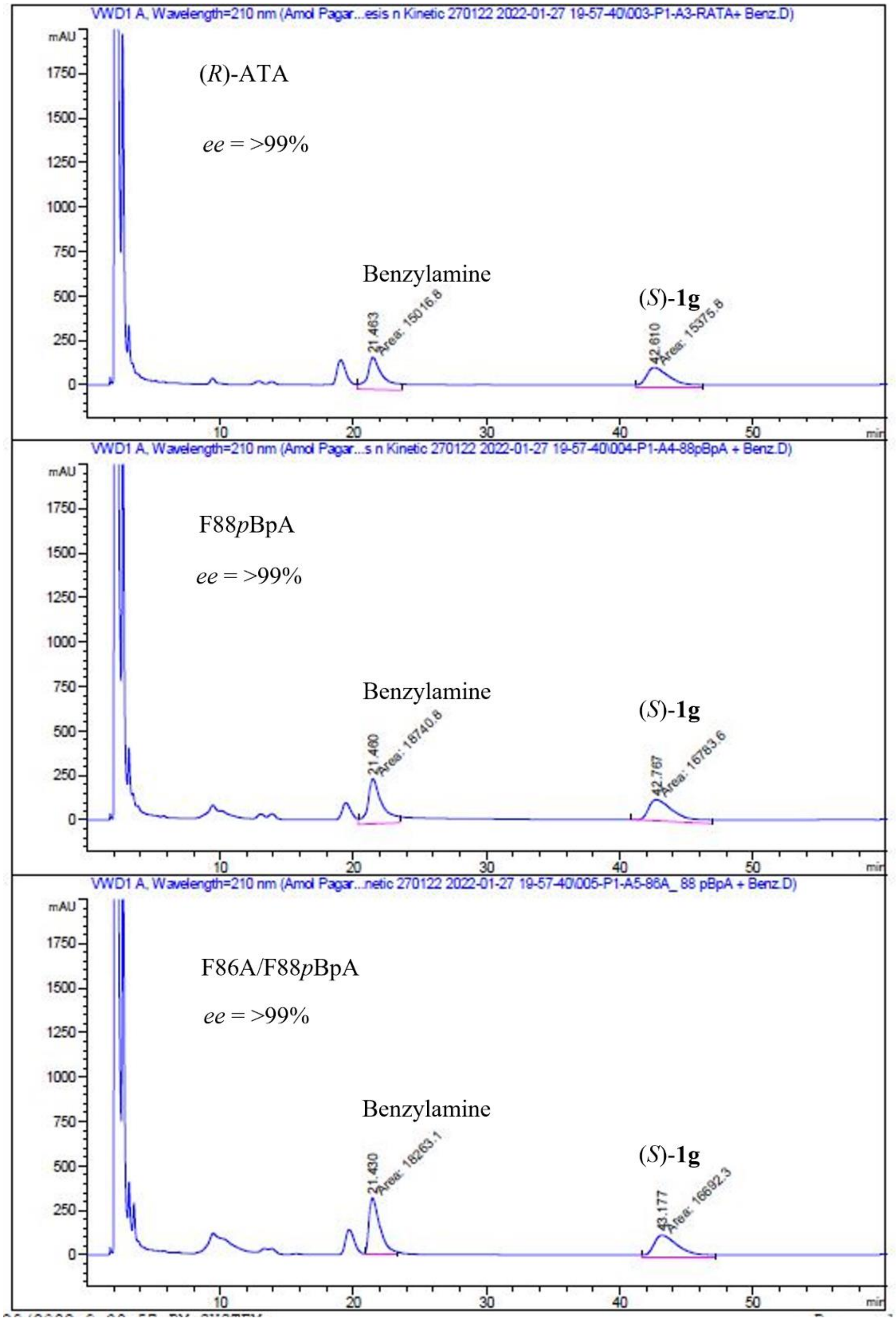


**Figure S7:** HPLC chromatogram of the kinetic resolution of 10 mM *rac*-**1g** using (*R*)-ATA and its variants with benzaldehyde as amino acceptor.

## References

Chin, J. W., Martin, A. B., King, D. S., Wang, L., and Schultz, P. G. (2002). Addition of a photocrosslinking amino acid to the genetic code of Escherichia coli. *Proc. Natl. Acad. Sci. U. S. A.* 99, 11020–11024. doi:10.1073/pnas.172226299.

Lee, Y. J., Schmidt, M. J., Tharp, J. M., Weber, A., Koenig, A. L., Zheng, H., et al. (2016). Genetically encoded fluorophenylalanines enable insights into the recognition of lysine trimethylation by an epigenetic reader. *Chem. Commun.* 52, 12606–12609. doi:10.1039/c6cc05959g.

Miyake-Stoner, S. J., Refakis, C. A., Hammill, J. T., Lusic, H., Hazen, J. L., Deiters, A., et al. (2010). Generating permissive site-specific unnatural aminoacyl-tRNA synthetases. *Biochemistry* 49, 1667–1677. doi:10.1021/bi901947r.

Park, J., Lee, Y., Ko, B. J., and Yoo, T. H. (2018). Peptide-Directed Photo-Cross-Linking for Site-Specific Conjugation of IgG. *Bioconjug. Chem.* 29, 3240–3244. doi:10.1021/acs.bioconjchem.8b00515.
